# Modified Sepiolite Nanoclays in Advanced Composites for Engineering Applications

**DOI:** 10.1021/acsanm.4c03115

**Published:** 2024-08-13

**Authors:** Yue Tang, Dankun Yang, Valeska P. Ting, Ian Hamerton, Jeroen S. Van Duijneveldt, Sébastien Rochat

**Affiliations:** † School of Chemistry, University of Bristol, Cantock’s Close, Bristol BS8 1TS, U.K.; ‡ School of Electrical, Electronic and Mechanical Engineering, University of Bristol, Queen’s Building, University Walk, Bristol BS8 1TR, U.K.; § Research School of Chemistry, Australian National University, Canberra ACT 2601, Australia; ∥ Bristol Composites Institute, School of Civil, Aerospace, and Design Engineering, University of Bristol, Queen’s Building, University Walk, Bristol BS8 1TR, U.K.; ⊥ School of Engineering Mathematics and Technology, University of Bristol, Ada Lovelace Building, Tankard’s Close, Bristol BS8 1TW, U.K.

**Keywords:** sepiolite, organic modification, microporosity, benzoxazine, clay−polymer nanocomposites

## Abstract

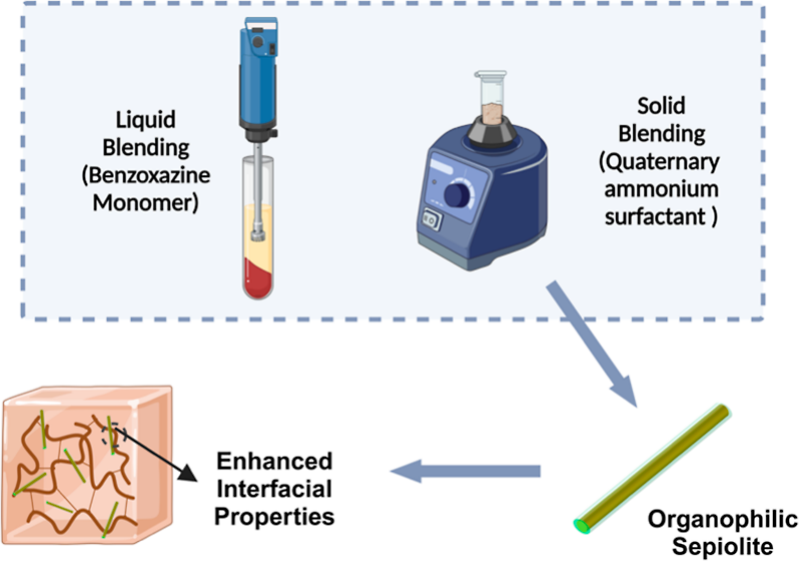

Clay–polymer nanocomposites (CPNs) containing a small weight fraction of nanoclay are known to display enhanced mechanical and thermal properties compared to neat polymers. However, the preparation and application of such nanocomposites remain challenging owing to the difficulties in dispersing nanoclays in polymer matrices. This study focuses on two surfactant-modified organophilic sepiolite clays to demonstrate the simplicity of the modification process, as well as on the use of a benzoxazine monomer (i.e., a CPN matrix precursor) itself as the modifier. Our in-house modified bespoke sepiolites achieve much better dispersion in a benzoxazine matrix, compared to the pristine clay, revealing their potential for applications as nanoenhancers for advanced composites.

## Introduction

The history of clay–polymer nanocomposites (CPNs) dates back to the late 1980s and has blossomed over the last 3 decades.^1−5^ Recently, many works have proven that the incorporation of clays can enhance various properties of polymers, including mechanical, thermal, flame retardancy, gas-barrier properties, etc. Such materials have been applied in automotive industries and are also promising for engineering applications in various industries, such as food packaging, biomedical, wastewater treatment, and so on.^6^ However, in most of the CPNs, the enhancer selected is a platelet-shaped clay, such as montmorillonite or kaolinite, and the incorporation of fibrous clays, such as sepiolite, has seldom been investigated.

**Figure 1:**
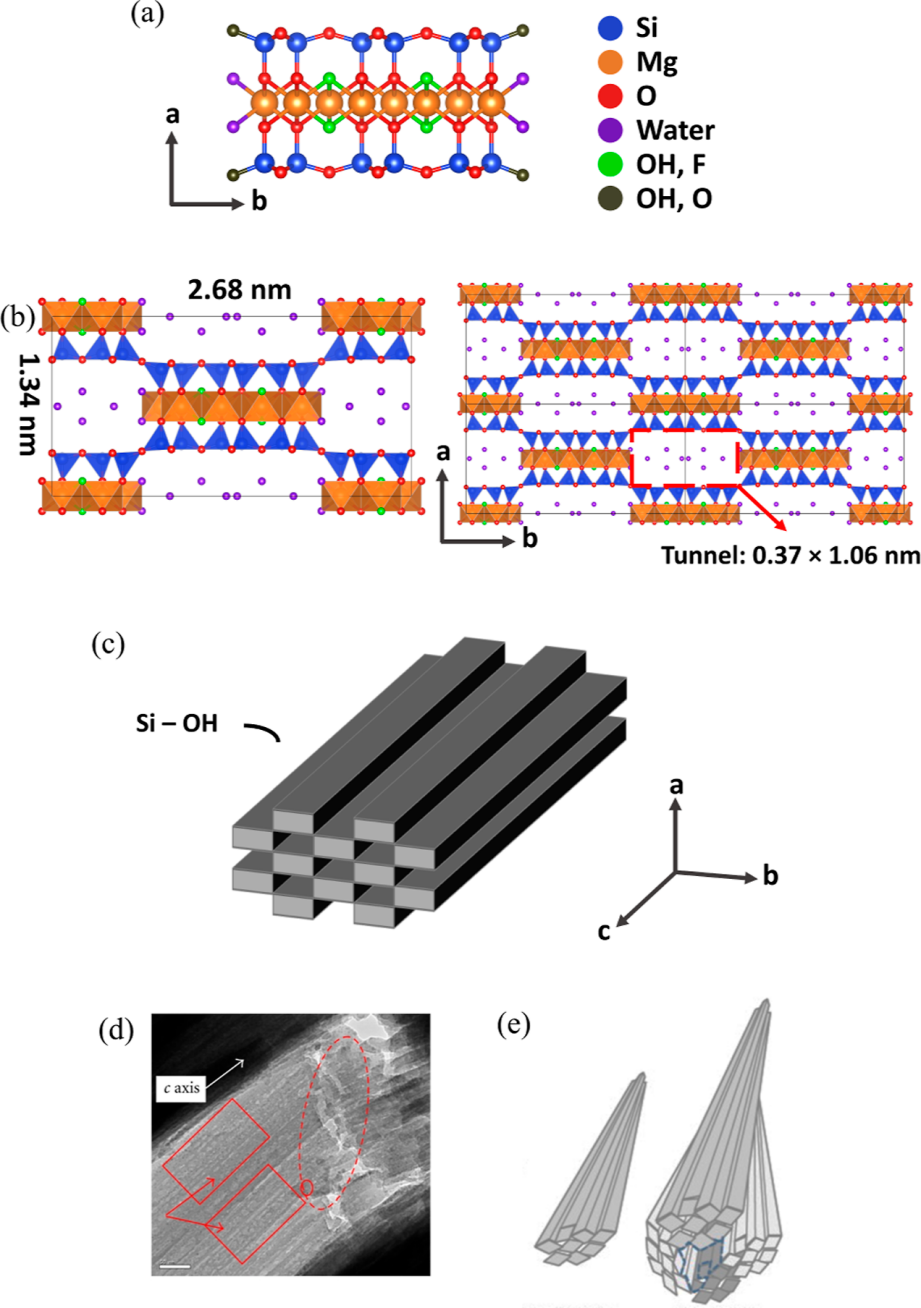
Hierarchical structures of sepiolite (a) T–O–T elemental block; (b) crystal structure of a basic unit and several repeated units, drawn using VESTA;^12^ (c) ideal crystal of sepiolite in the form of a lath (single crystal), which is the smallest primary unit that can be observed; and (d) crystal defects along the *c*-axis, adapted from Tang et al.^10^ Copyright 2012 Tang et al.; (e) rod (left) composed of several sepiolite laths and bundle (right) composed of several rods, reproduced with permission from Suárez and García-Romero.^9^ Copyright 2012 Elsevier B.V. For the specific definition of lath, rod, and bundle, please refer to García-Romero and Suárez.^13^

Sepiolite, a clay-like mineral, is a hydrated magnesium silicate with a hierarchical structure (Figure 1). The basic elemental block of the sepiolite mineral comprises a sheet of [MgO_6_] octahedra (O) inserted between two sheets of [SiO_4_] tetrahedra (T) (Figure 1a). Coordinated water molecules are bonded to the Mg^2+^ cations, which are at the edges of the O sheets. Moreover, the hydroxide ions linked to the Mg^2+^ are sometimes substituted by fluoride ions due to the similarity of OH^–^ and F^–^ in both electronegativity and ionic radius, giving sepiolite a unit cell formula of Si_12_O_30_Mg_8_(OH,F)_4_(H_2_O)_4_·8H_2_O.^7^ The basic crystal structure is shown in Figure 1b, with dimensions of *a* × *b* × *c* = 1.34 nm × 2.68 nm × 5.28 nm.^8^ The formed cavities, showing a cross-section dimension of 1.06 × 0.37 nm, are filled with zeolitic water, which is associated with the coordinated water by hydrogen bonding. The blocks and cavities extend in the *c*-axis direction with Si–OH (silanol) groups existing on the external surface, forming long sepiolite laths and fiber-direction tunnels (Figure 1c). Such structural tunnels provide sepiolite with intracrystalline microporosity.^9^ Some crystal defects are also formed, leading to discontinuation of the *c*-axis crystal plane, as well as offering some extra characteristic radial pores with other sizes (Figure 1d).^10^ Moreover, rods and bundles are formed with the aggregation of the sepiolite laths, offering sepiolite with interfiber micro-, meso-, and even macro porosity (Figure 1e).^9^ The pore sizes of these three levels of porosity are defined clearly as follows: micropores are smaller than 2 nm, mesopores are between 2 and 50 nm, and macropores exceed 50 nm.^11^

An individual sepiolite lath can show typical particle lengths ranging from 500 to 1500 nm and diameters from 20 to 40 nm.^14^ Such needle-like shapes make it easier to achieve a higher degree of dispersion compared to commonly used platelet clays, for example, montmorillonite,^2^ as the latter possess a relatively higher surface-area-to-volume ratio (SA/V), which results in significant van der Waals interactions between monolayers.^15^ Although the hydrophilic nature of sepiolite is a potential issue limiting its dispersion in hydrophobic matrices,^16^ this can be mitigated using surface treatment methods.^17,18^ The high concentration of surface silanols present along the length of the sepiolite particle makes it easy to perform coupling reactions with organic surfactants, as well as to exploit other interactions such as hydrogen bonding and van der Waals interactions for adsorption.^16^ In addition, inorganic cations of the sepiolite allow for ion exchange, for example with organic ammonium ions, further increasing the scope of potential modifications.^18,19^

Tailoring sepiolite functionalities allows the clay particles to be more compatible with organic polymer matrices. Here, benzoxazine is selected as an example due to its unique combination of advantages, including exceptionally near-zero polymerization shrinkage, low water adsorption characteristics, and moderately high glass transition temperatures.^20^ Following the first reports of the polymerization of the benzoxazine molecule in 1944,^21^ the development of polyfunctional benzoxazine resins by Ning and Ishida heralded the potential of this material.^22^ With rapid development in the past 3 decades, polybenzoxazine (PBz) resins are now regarded as attractive alternatives to traditional epoxy and phenolic resins. While their near-zero shrinkage and low water adsorption bring the material benefits of high-precision dimension and stable performance in demanding applications (e.g., aerospace and offshore industries), there is still a major obstacle to their wider adoption, namely, their inherent brittleness. The incorporation of nanoclays has proven to be an effective method to toughen thermoset resins, and it has been shown that even a small weight fraction of clay (<5 wt %) can increase the fracture toughness (*K*_Ic_) of resin matrices by over 50%.^1,23,24^ To our knowledge, there is only one study reporting the formation of PBz/sepiolite nanocomposites, and the utilization of the pristine sepiolite leads to a poor dispersion with large aggregations.^25^ Therefore, a stronger interfacial contact between sepiolite and matrix, as well as an excellent dispersion, are highly desired to achieve an optimal enhancement of mechanical properties.

In this study, three surface-modified sepiolites were investigated as highly effective toughening fillers for a PBz matrix: one was a commercial organophilic clay (PANGEL B20) and the other two (surfactant-modified clay, SMC, and benzoxazine-modified clay, BMC) were obtained by modifying a pristine sepiolite in-house. SMC is functionalized by a commonly used ammonium salt surfactant using facile solid blending, while BMC is modified using a monobenzoxazine utilizing solvent blending. Few cases have been reported where matrix monomers are used to functionalize nanosized enhancers, and the morphology of such modified nanoparticles is not discussed in detail.^26−28^ Here, the detailed composition, thermal properties, and structure of these organophilic sepiolites were assessed and compared with those of pristine clay. The dispersion of our modified sepiolites in a benzoxazine matrix was also investigated with the ultimate aim of promoting the application of nanoclay in engineering advanced composites.

## Materials and Methods

### Materials

Pristine sepiolite and benzyldimethyltetradecylammonium chloride dihydrate >98% were obtained from Sigma-Aldrich. Tetrahydrofuran (THF) ≥ 99.5% and ethanol ≥99.8% were purchased from Fisher Scientific. Organophilic sepiolite PANGEL B20 was kindly offered by Tolsa (Madrid, Spain). Benzoxazine Araldite MT 35500 (hereafter CA-a) was kindly provided by Huntsman Advanced Materials (Basel, Switzerland). Both the pristine and commercial (PANGEL B20) sepiolites are nanosized rod-like particles.

All materials were used as received without any purification.

### Preparation of Organo-Modified Sepiolites

**Figure 2:**
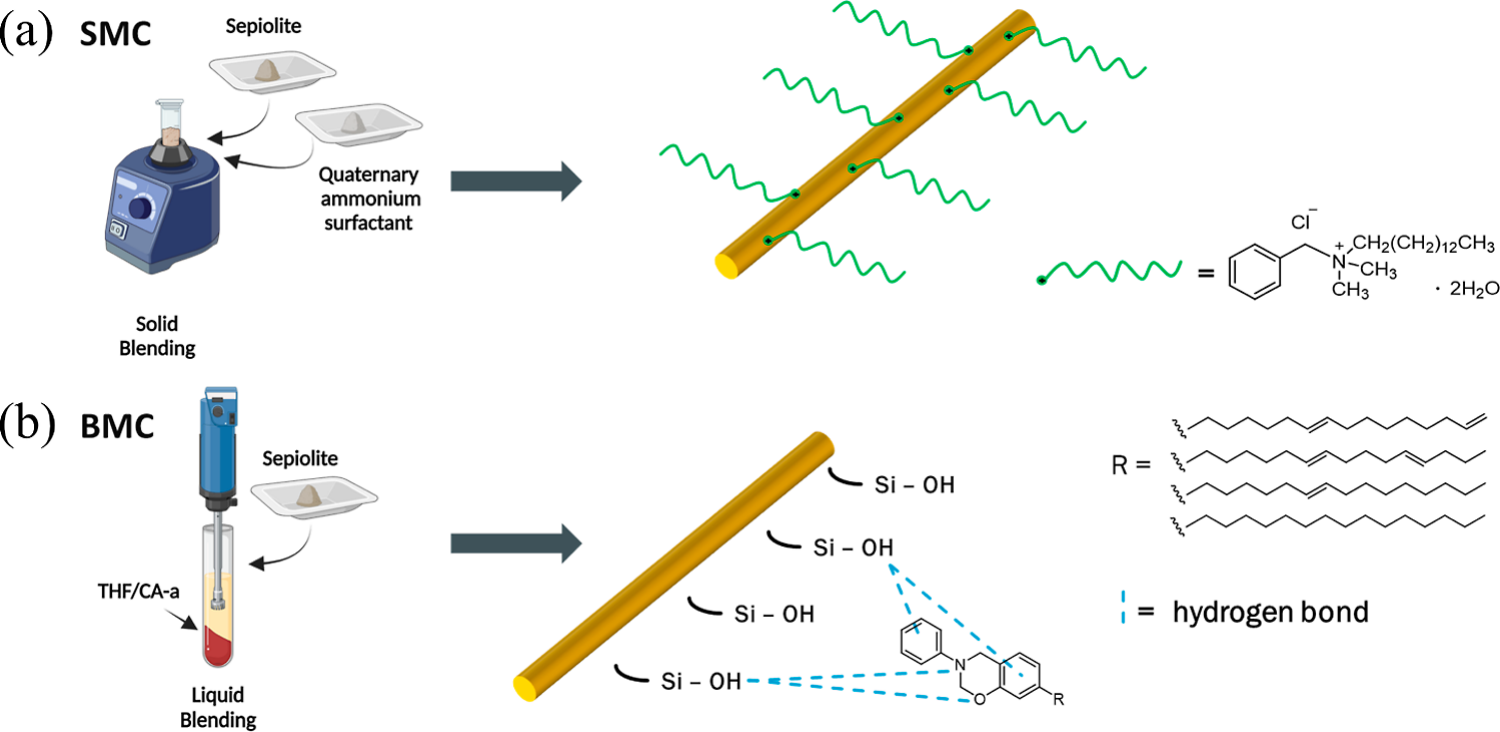
Schematic of the modification process and mechanism for (a) SMC and (b) BMC.

Two in-house-modified clays containing 10 wt % of modifiers were prepared in this study (Figure 2): a clay functionalized using benzyldimethyltetradecylammonium chloride dihydrate (C_23_H_42_ClN·2H_2_O) was made by solid blending, using metal balls and an IKA Ultra TURRAX Tube Drive at 6000 rpm for 10 min. The ammonium salt-modified clays (hereafter SMC) thus obtained were used without any further purification.

The second clay (hereafter BMC) was modified by using monofunctional benzoxazine CA-a. The solvent blending method was selected for the BMC preparation, and IKA Ultra TURRAX T-18 was utilized in the process. Pristine clay and CA-a were dispersed in water and THF, respectively, at 5 wt % with 10–15 mL solvent, mixing for 5 min at 20,000 rpm. The clay/water dispersion and the CA-a/THF solution were mixed and stirred for another 10 min at 20,000 rpm (weight ratio of clay/water: CA-a/THF was 9:1). The resulting blend was filtered and washed with deionized water to yield powders that were finally left in a Sheldon Shel Lab 1445 vacuum oven at a temperature of 60 °C under vacuum until fully dried.

The modification processes were carried out at room temperature. The formulations of these sepiolites, as well as the information on the corresponding surfactants, are listed in Table S1. Typical batch sizes obtained with our methods were 1.0 g for SMC and 0.5 g for BMC.

### Elemental Analysis

The clays and surfactants were characterized by elemental analysis to determine the contents of carbon, hydrogen, and nitrogen, as well as the weight fraction of the modifiers in the final clay product. Moreover, the modification quality and stability of the in-house-modified clays SMC and BMC were tested using an additional washing step, performed as follows: modified clay samples (approximately 0.1 g) were suspended in THF (100 g, 113 mL) and mixed with a magnetic stir bar for 1 h. The suspensions were then filtered over filter paper (3 μm particle retention, Whatman).

Samples were dried under a vacuum at 40 °C for 6 h and tested at OEA Laboratories Ltd. (Exeter, UK).

### Thermogravimetric Analysis

A TA Instruments thermogravimetric analysis (TGA) Q500 was used to carry out the TGA characterization, determining the thermal stability and char yield differences of samples. Platinum pans were used with the powdered sample weighing around 10 mg. The testing was conducted with a heating rate of 10 °C min^–1^ and a heating range of 25–750 °C in a nitrogen flow (60 mL min^–1^).

### Fourier-Transform Infrared Spectroscopy

FTIR was performed using a PerkinElmer Spectrum Two FTIR spectrometer with a universal attenuated total reflection accessory. Samples were analyzed directly, and 16 scans were acquired in the wavenumber range of 4000–400 cm^–1^ at a resolution of 1 cm^–1^. Transmittance was analyzed and normalized using Origin. Neither baseline correction nor smoothing were applied to the obtained data.

### Transmission Electron Microscopy–Energy-Dispersive X-ray Spectroscopy

The clay particles were dispersed in ethanol with a weight fraction of 0.05 wt % and then dried on a holey carbon grid for observation. Subsequently, samples were coated with graphite (Agar Scientific, UK) and imaged using a field-emission gun JEM-2100F (JEOL, Japan) at 200 kV, equipped with an Orius SC1000 camera (Gatan, US). The micrographs were collected in the scanning transmission electron microscopy mode, while elemental composition data were collected using an X-Max 80 mm^2^ EDX detector and analyzed with AZtec software (Oxford Instruments, UK).

### X-ray Diffractometry

The powder X-ray diffraction (PXRD) patterns were collected by using a Bruker D8 Advance powder X-ray diffractometer in a flat plate geometry with Cu radiation (wavelength of 1.54 Å) equipped with an Oxford Cryosystems PheniX stage. Scans were recorded over the 2θ range 5–50°, with a step size of 0.02° 2θ and a scanning rate of 1.2° min^–1^.

### Gas Adsorption

The CO_2_ and N_2_ adsorption–desorption isotherms were measured on clays using a Micromeritics 3-Flex volumetric gas sorption analyzer from 0 to 1.0 bar at 273 K (ice bath) and 77 K (liquid N_2_), respectively. All sepiolite powders (ca. 100 mg) were degassed at 90 °C under high vacuum (10^–5^ mbar) for 15 h prior to sorption measurements. The obtained isotherms were analyzed using embedded MicroActive software.

### Optical Microscopy

To evaluate the dispersion of different sepiolites in a benzoxazine matrix, clay particles were incorporated into CA-a benzoxazine by the following method: tetrahydrofuran (THF) was first mixed with CA-a with a weight ratio of 7:3 using an IKA Ultra TURRAX T-18 high shear mixer with a speed of 20,000 rpm for 5 min. Subsequently, the different sepiolite powders (weight ratio of clay: CA-a = 1:10) were added to the THF/CA-a blend for another 5 min of mixing. The obtained mixtures (THF/CA-a/sepiolite) were added as a droplet to a microscope slide and observed using an Olympus BX51 Microscope in the polarizing mode.

ImageJ was used to determine the number of large clusters in each sample.^29^

## Results

### Preparation and Characterization of Modified Clay Samples

#### Outcome of the Clay Modification Procedures

The results of the elemental analysis of clays and modifiers are shown in Table 1, where values are calculated from the average of the duplicate tests. The high mass percent of carbon and increased fraction of nitrogen in modified clays are strong evidence of the presence of surfactants. As the weight fraction of carbon in modifiers is larger than that of nitrogen, the former was used to calculate the weight fraction of the modifier in the in-house functionalized clays (SMC and BMC). The C, H, and N amounts measured experimentally are generally consistent with the predicted values.

**Table 1:** Elemental Analysis Results of Modifiers and Sepiolites^a^

	modifiers		sepiolites					
	ammonium salt	benzoxazine CA-a	pristine clay	PANGEL B20	SMC	BMC	washed SMC	washed BMC
Experimental Mass Percent (%)								
C	71.84 (0.225)	82.31 (0.065)	0.38 (0.010)	9.46 (0.020)	7.74 (0.025)	9.54 (0.030)	6.39 (0.020)	8.60 (0.800)
H	11.82 (0.010)	9.50 (0.005)	1.85 (0.025)	2.47 (0.010)	2.33 (0.015)	2.29 (0.030)	2.17 (0.025)	2.32 (0.145)
N	3.68 (0.020)	3.39 (0.020)	0.00	0.38 (0.005)	0.35 (0.005)	0.32 (0.005)	0.25 (0.005)	0.18 (0.005)
Theoretical Mass Percent (%)								
C	75.06	83.00	0.00		7.53	8.57	≤7.53	≤8.57
H	11.50	9.85	1.05		2.85	2.61	≤2.85	≤2.61
N	3.68	3.35	0.00		0.37	0.34	≤0.37	≤0.34
Total Organic Content (wt %)								
					10.30	11.18	8.41	10.03
Total Organic Treatment Applied (wt %)								
					10.00	10.00	≤10.00	≤10.00

a Theoretical mass percent calculated based on the weighted arithmetic mean of C, H, and N values of pure sepiolite and corresponding modifier with a weight ratio of 9:1. The data in brackets are standard deviations calculated from duplicate tests.

Moreover, both SMC and BMC still show high organic contents after washing, which is encouraging evidence that the surfactant is reasonably bound to the clay surface. From a mechanistic perspective, in BMC, CA-a benzoxazine is physisorbed on the clay surface via hydrogen bonds (Figure 2). The sepiolite itself can adsorb a lot of CA-a even without any mixing (Figure S1). During the process, Si–OH groups act as neutral adsorption sites, of which the upper limit is about 0.60 mmol g^–1^.^30^ There is approximately 0.24 mmol g^–1^ of surfactant on the washed BMC, which is still far below the theoretical limit. In the case of SMC, both neutral and charged adsorption sites are available to bind to the ammonium species within the surfactant molecules. The cation exchange capacity (CEC, in the range of 0.10–0.15 mmol g^–1^) can limit the amount of surfactant, which is strongly adsorbed.^30^ The amount of ammonium salt left on washed SMC is about 0.25 mmol g^–1^, which is somewhat higher than the CEC value. The additional surfactant molecules may be connected to the neutral adsorption sites, or attached to the first layer of surfactant, instead of the sepiolite directly. The higher surfactant adsorption values compared to the CEC value are commonly seen. For example, Lemic et al.^18^ reported that the adsorbed amounts of several quaternary amines can be over 250% of the CEC of sepiolite, and some other researchers found that the adsorbed amounts of divalent organic cations can get above 4-fold the CEC.^30^ It should be noted that, although the CEC value is quoted here, the modification process of SMC should be considered as a solid–state reaction instead of a classic solvent-based cation exchange reaction and that ion–dipole interactions (i.e., adsorption) also play a role in the process in addition to the exchange of cations.^31^

Looking further at the modification procedure, for BMC, a high loading of sepiolites is dispersed in water under high shear, with individual needle-like clay particles being dragged out from the bundles and leading to a 3D network.^17,32^ Such a stable water/sepiolite aqueous gel (Figure S2) allows the THF/CA-a solution to mix thoroughly with individual sepiolite particles, while the solid blending used for SMC does not allow such an intimate mixture between the sepiolite and surfactant. This difference might explain why fewer modifiers can be washed out of BMC compared to SMC. However, the much easier fabrication process of SMC might offer extra advantages for industrialization. Moreover, the absence of solvents in organophilic sepiolite manufacture is considered to be more environmentally friendly, especially in large-batch manufacture.

**Figure 3:**
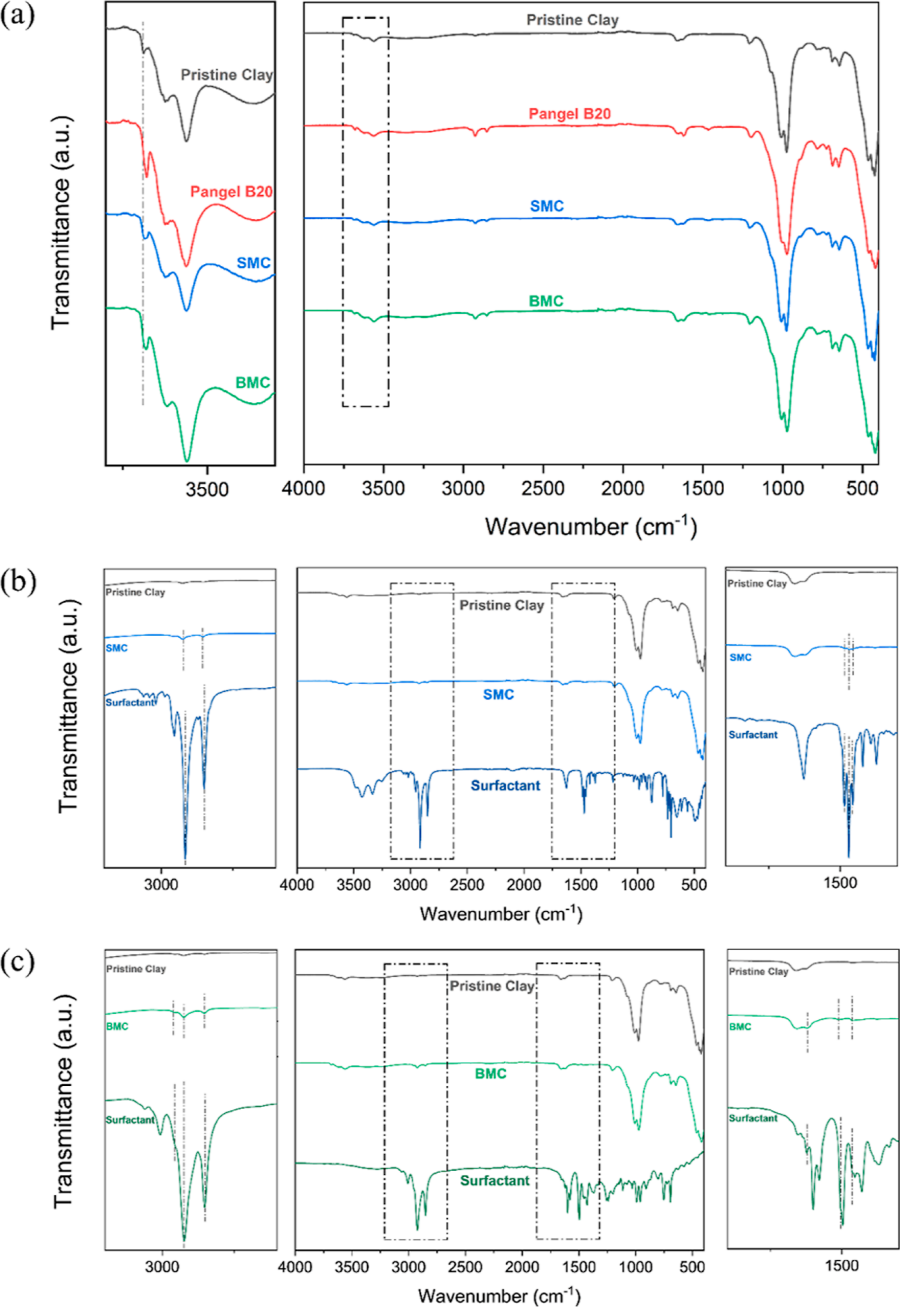
FTIR spectra (shown as transmittance as a function of wavenumber) of (a) four different clays offset vertically for comparison, with an enlargement of the range (3800–3300) cm^–1^. Transmittance is normalized based on the Si–O–Mg vibration bands (at 424 cm^–1^). (b) SMC in blue, pristine clay in black, ammonium salt surfactant in dark blue, and (c) BMC in green, pristine clay in black, and CA-a surfactant in dark green, with an overview range of (4000–400) cm^–1^, an enlargement of the range (3200–2600) cm^–1^, and an enlargement of the range (1900–1300) cm^–1^.

The FTIR spectra (acquired between 4000 and 400 cm^–1^) of the samples are shown in Figure 3a. All specimens show intensive characteristic bands around 1000 cm^–1^, indicating the Si–O stretching in the tetrahedral sheet.^33^ However, the spectra of the three functionalized sepiolites show obvious changes around 3000 and 1500 cm^–1^ compared to the pristine clay, which illustrates the incorporation of organic components. Meanwhile, some slight changes can be observed in the spectral range (3800–3300 cm^–1^): the 3689 cm^–1^ bands related to the Mg_3_OH interior of the crystal block are perturbed somehow in all the modified sepiolites, which might be related to the surfactants absorbed to the crystal defects, acting as physical hindrances.^34,35^ Moreover, in the spectral fingerprint region (1300–400 cm^–1^) reflecting the lattice vibrations related to Si–O, Si–O–Mg, and Mg–OH,^33^ all samples show similar results with negligible differences, which could reflect that the modification of the clay does not distort its crystal structure but only affects the texture and morphology of these particles.^36^

Figure 3b,c offer more information about how the selected modifiers change the spectra of SMC and BMC, respectively. For the former, in the 1900–1300 cm^–1^ region, the new bands can be attributed to the C–H bending of the methylene and methyl groups of the surfactant. In the 3200–2600 cm^–1^ spectral region, the additional bands correspond to C–H stretching. For the latter, in the 1900–1300 cm^–1^ spectral region, the new bands are assigned to the C=C stretching and C–H bending modes in the side chain of CA-a. In the 3200–2600 cm^–1^ spectral region, the observed new bands are dominated by the C–H stretching.^37^

#### Thermal Properties

**Figure 4:**
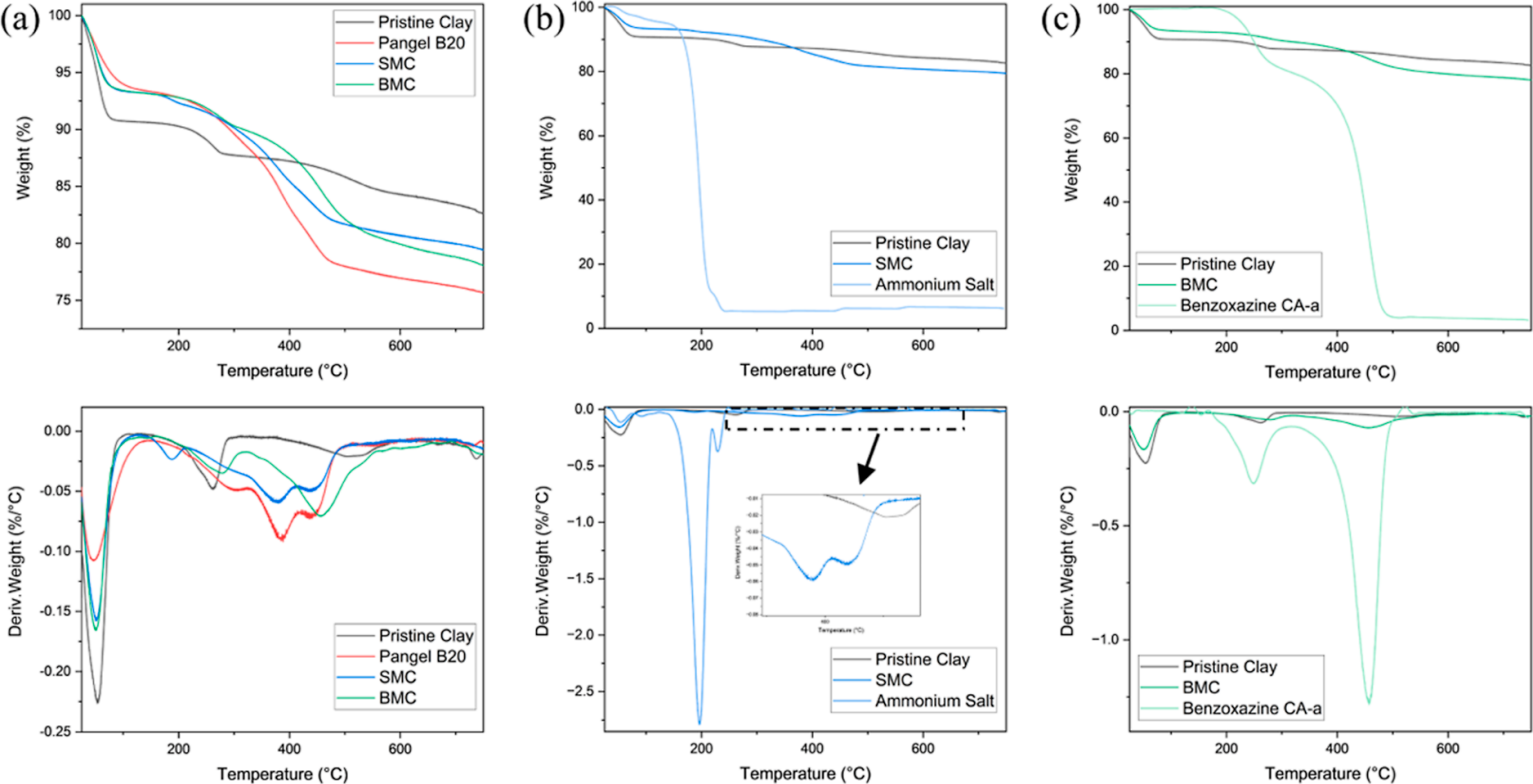
Thermogravimetric analysis results of four clays and the corresponding surfactant in a nitrogen atmosphere with a ramping rate of 10 °C min^–1^. First row and second row refer to the TGA and DTG curves, respectively. DTG curves are smoothed using Origin for noise removal. (a) Four clays; (b) laboratory-prepared SMC compared with pristine clay and surfactant; and (c) laboratory-prepared BMC compared with pristine clay and surfactant.

TGA offers information about sample mass loss versus temperature changes. The TGA and differential thermogravimetry (DTG) curves of all samples are shown in Figure 4. Generally, for the native clay, four distinct phases of mass loss could be distinguished, which can be attributed to the loss of hygroscopic water, hydration water, coordination water, and hydroxyl water, respectively.^33,38^ For the first step of the degradation (25–150 °C), all modified clays show lower mass losses compared to the pristine clay, which is mainly due to the surfactant layer coverage preventing water adsorption.^18,32^ For the second and third steps of the degradations (150–610 °C), all organophilic sepiolites show significantly higher mass losses, which should be related to the degradation of organic modifiers. For the final step of the degradation (>610 °C), all the clays show similar behavior.

Figure 4b,c offer details about how the incorporation of different modifiers affects the thermal stability of the sepiolite. Generally, BMC shows a DTG curve that matches the shape of the combination of pristine clay and benzoxazine CA-a, while SMC differs as the significant decomposition of ammonium salt, originally centered around 200 °C, is partially shifted to around 400 °C in SMC, which may indicate some of the surfactant molecules (or part of their side chains) are inserted into the relatively large crystal defects or inner channels of sepiolites, preventing them from an earlier decomposition.

#### Microstructure

**Figure 5:**
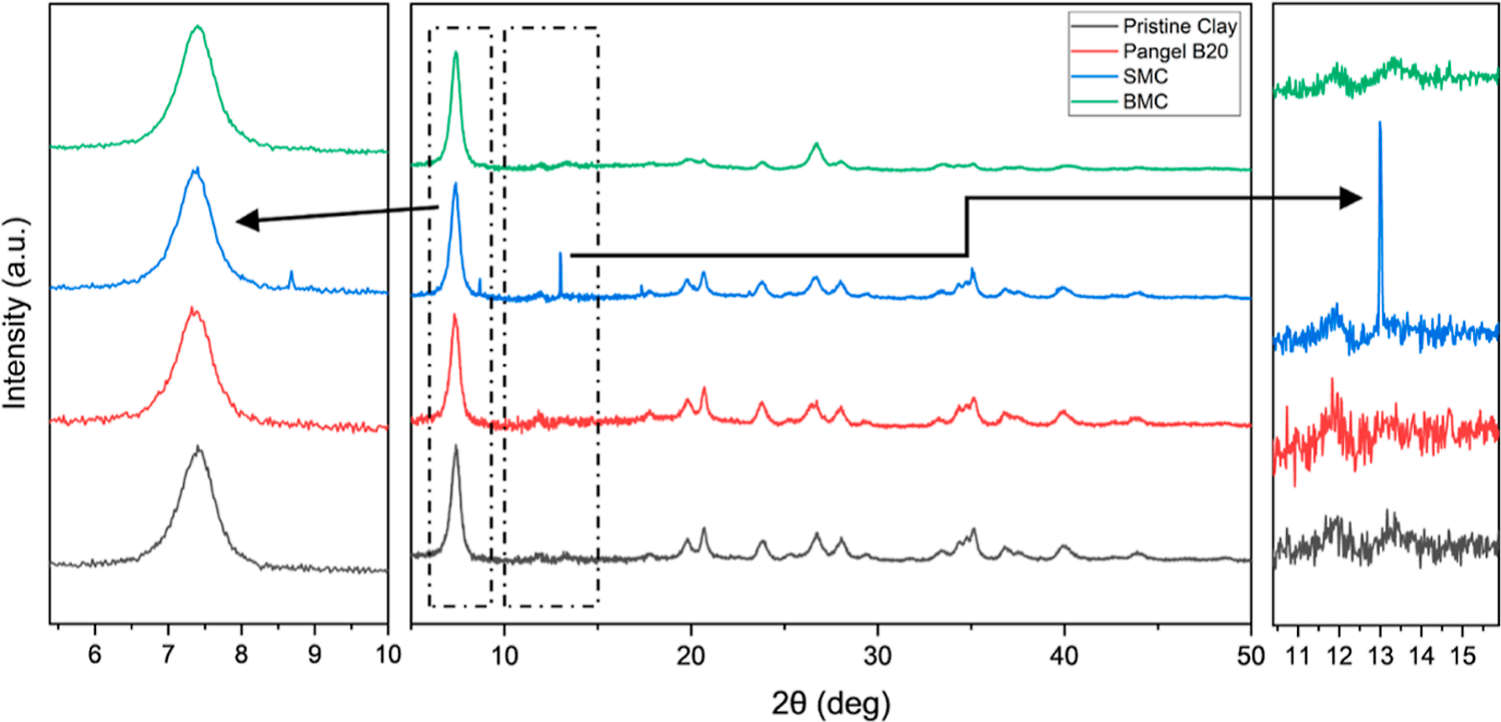
PXRD patterns of sepiolites, normalized to the (110) peak, the right enlarged picture focused on the (110) peak, and the left enlarged picture focused on (130) and (040) peaks.

Some obvious differences between modified and unmodified sepiolites are observed in the PXRD patterns (Figure 5). Owing to the overlapping data and the relatively low diffraction, only the peak centers of (110) and (130) planes were recorded. Meanwhile, the *d*-values were calculated based on Bragg’s law (Table S2).^39^ All the samples show comparable *d*-values with theoretical values, of which (110) is calculated as 11.985 Å and (130) was calculated as 7.433 Å using VESTA.^12^ Generally, SMC shows the most different pattern with some intense additional peaks. This indicates the sample in all likelihood contains some surfactant in crystalline form (Figure S3), which may be explained by the following two reasons: first, the surfactant may be well ordered on the clay surface and second, there may be a slight excess of (unbound) surfactant due to the lack of a washing process. Meanwhile, PANGEL B20 shows slightly shifted diffraction peak centers compared to the other three samples, which is not surprising, as this sepiolite came from a separate source, whereas SMC and BMC were prepared from the same pristine sample. Del Rio et al. pointed out that the *d*-value differences are related to the cell originated by compositional variations caused by isomorphic substitutions.^40^ Although there are some variations among those samples, all of the clays show characteristic diffraction peaks indicating no significant change in the crystalline structure.

**Figure 6:**
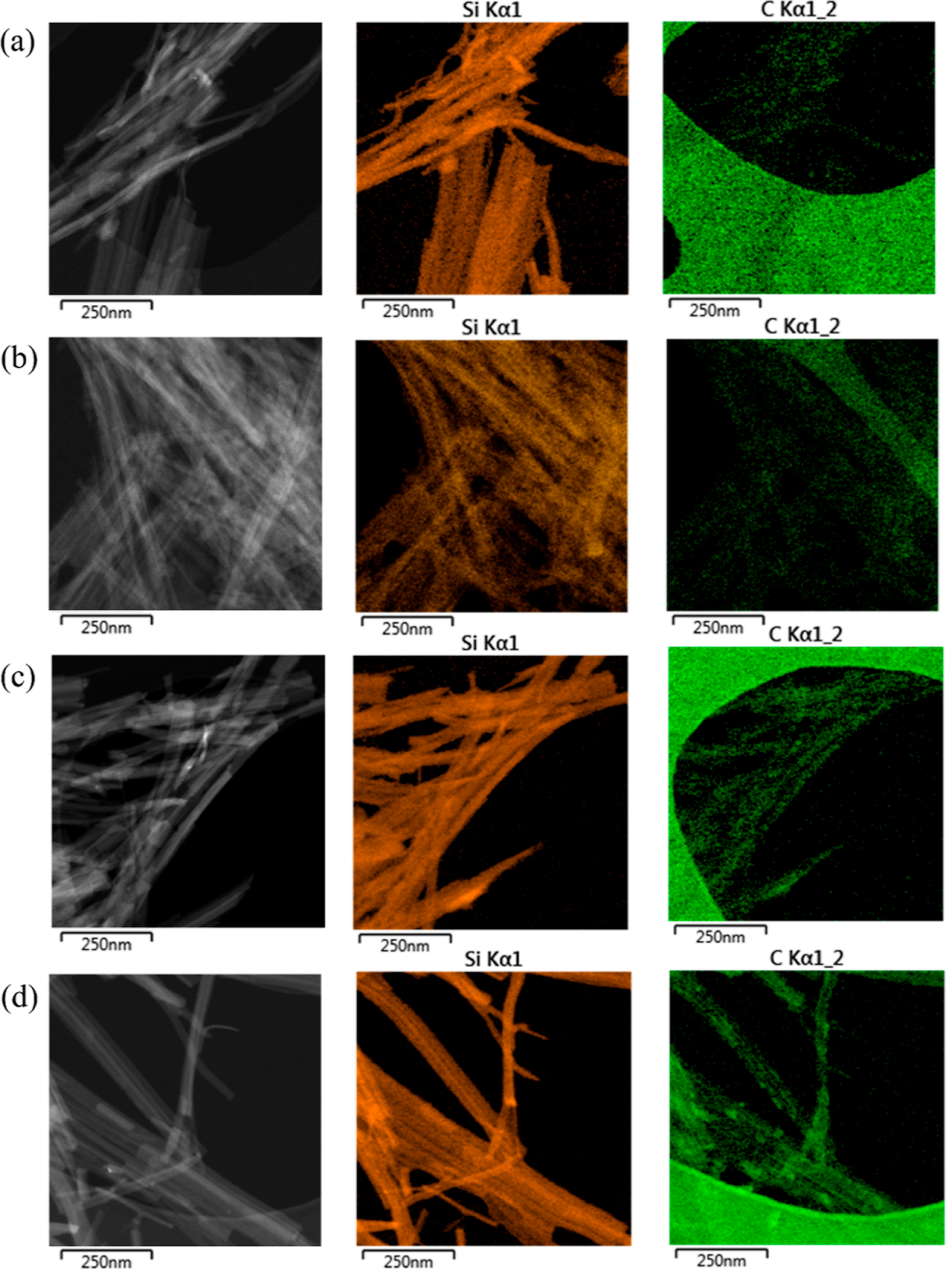
TEM–EDX data for the four different sepiolites on a holey carbon grid (a) pure sepiolite, (b) PANGEL B20, (c) SMC, and (d) BMC.

In this study, the uniform organic layers on clay rods are convincingly visualized by TEM–EDX, while an alternative method might utilize high-resolution TEM analysis, as reported by Chen et al.^41^Figure 6 shows the elemental mapping of these four different sepiolites in which Si was used as the characteristic element for the sepiolite, while C was selected as the characteristic element for the surfactants. All the clays show highly concentrated Si reflecting a clear outline of the sepiolite particles, which is in line with expectations, as Si is the main component of the sepiolite. As for the surface distribution of carbon, pure clay shows some very vague patterns, which might be attributed to the coating and impurities, while for PANGEL B20, a slightly clearer shape could be observed. For the SMC and BMC, the contour of sepiolite is much more distinct. Interestingly, in the BMC micrograph, carbon seems to be concentrated more in the outer contour line of the clay. Generally, the carbon elemental mappings prove the organic coating appears evenly distributed on the functionalized clays. Also, the organic layer does not extend noticeably within the mineral core, indicating a very thin layer of the modifier while the clay particle underneath remains intact. For a general particle size investigation using TEM, readers can refer to Table S3.

**Figure 7:**
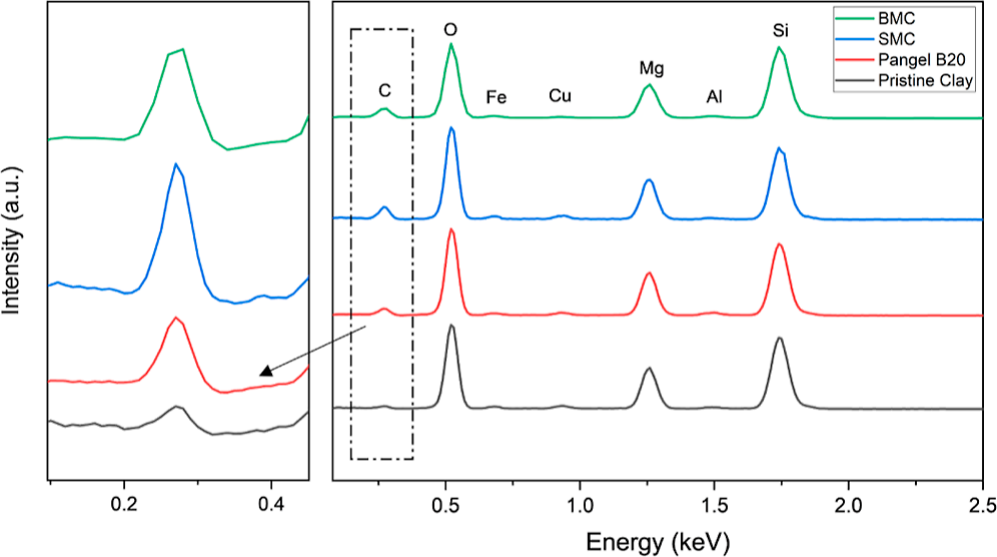
EDX spectra of clays, normalized using the Si peak, with an enlarged figure focused on the C signal.

The EDX spectra and elemental atomic weight values of these clays are shown in Figure 7 and Table S3. Generally, all the modified clays show much higher C At % compared to the pure clay, which is consistent with the elemental analysis, indicating the successful attachment of the surfactants. Moreover, although the EDX spectra confirm that the primary constituent elements of sepiolite are Si, Mg, and O, it is not surprising to see all the clay show Si At %: Mg At % values, which are somehow different from the ideal formulation. Sepiolite products always contain some impurities like free silica (quartz) and Illite, and while the former could lead to an increasing Si content, the latter can incorporate elements, such as K, Al, Mg, and Fe.^42^ Meanwhile, the substitution of Mg^2+^ by Al^3+^ and Fe^3+^ is also possible,^43,44^ and despite these imperfections, our data are comparable to literature values.^45^

**Figure 8:**
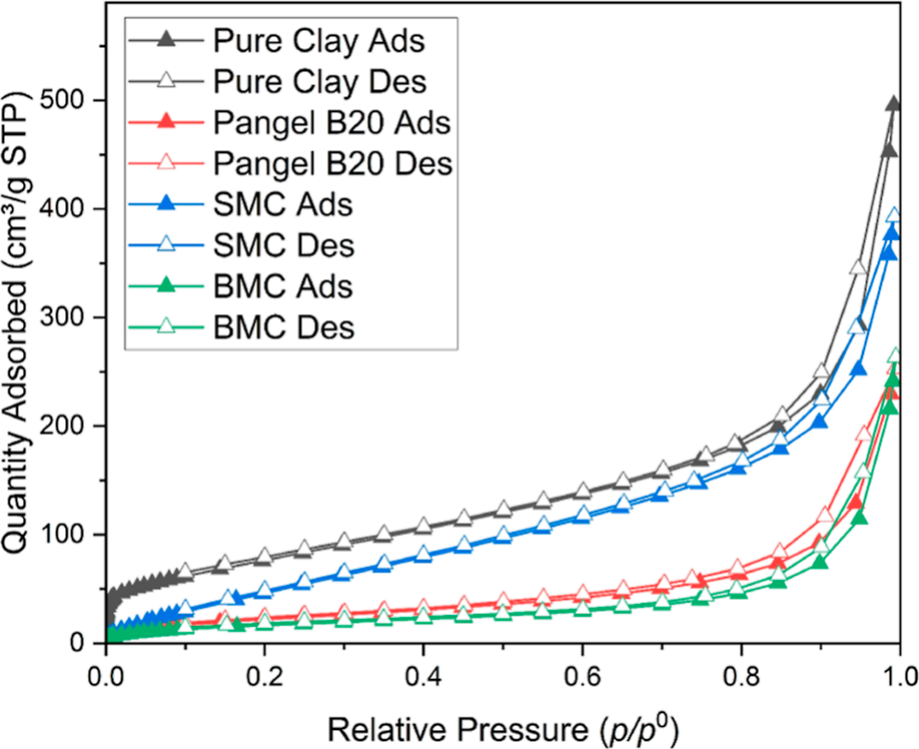
N_2_ gas adsorption at 77 K for the four sepiolites (solid triangles represent adsorption, and open triangles are desorption).

The N_2_ gas adsorption curves are shown in Figure 8. It is clearly seen that the pure sepiolite shows a much higher volume adsorbed compared to the modified samples, which may have two possible reasons: first, some cavities in the sepiolite may be large enough to be partially occupied by the modifier or its side chains, which limits the amount of gas that can be adsorbed.

A second reason could be that the modifier covers the outer surface of the sepiolite particles, filling the interparticle space and making the inner pores inaccessible to the nitrogen gas molecules. Moreover, the marked differences in the curve shapes of the adsorption/desorption isotherms give some indication about the pore size distribution as well as the hierarchical structure of the clays. The pure sepiolite shows a pattern which combines Type I(a), Type II, and Type IV(a) isotherms, according to the IUPAC classification.^46^ At extremely low *p*/*p*^0^ values (<0.01), a steep adsorption of gas happens, indicating micropores filled by N_2_. With the increase of pressure, the isotherm first shows a linear region indicating the formation of the multilayer gas coverage at medium *p*/*p*^0^ values on the mesopore walls. A sharp increase at higher pressure (*p*/*p*^0^ > 0.80) is then observed, which is related to the macropores in the sample, reflecting the unrestricted monolayer-multilayer adsorption in the high *p*/*p*^0^ range.^46^ Moreover, the pure sepiolite shows some hysteresis at high *p*/*p*^0^ values, identified as a Type H3 hysteresis loop,^46^ which can be attributed to nonrigid pores formed in the interparticle space generated by the arrangement of the particles.^47^

The detailed morphological data from the testing are given in Table 2. The specific surface area values (SSA_BET_) were determined using the Brunauer–Emmett–Teller (BET) method^48^ in the relative pressure (*p*/*p*^0^) range 0.05–0.3. The t-plot method^49^ was utilized to calculate the micropore surface area (SSA_μp_), micropore volume (*V*_μp,t_), as well as external surface area (SSA_Ext_), combining the thickness curve proposed by Harkins and Jura.^50^ The Barrett–Joyner–Halenda (BJH) method^51^ helps define the mesopore volumes (*V*_mp,BJH_) of different samples. The pure sepiolite shows the highest SSA_BET_ of 289 m^2^ g^–1^, which is comparable to the literature value 296 m^2^ g^–1^ and the typical measured values from ∼230 to ∼320 m^2^ g^–1^.^19,30^ Moreover, the native sepiolite shows reasonable mesopore and micropore volumes, which are of the same magnitude as literature values.^9,45^ When looking further at the SSA_BET_ values of the modified clays, it is not surprising to see such a dramatic decrease with ∼10 wt % surfactant addition. Mejía et al.^32^ reported a reduction of SSA_BET_ from 355 to 100 and to 50 m^2^ g^–1^, respectively, for sepiolite modified by ∼10 wt % poly(ethylene glycol) (PEG) and Vitamin E tocopherol poly(ethylene glycol) succinate (TPGS). The variations of SSA_BET_ reduction can be related to the molar mass of the nonporous surfactant molecule, as well as the final morphology of the sepiolite surface. For example, a continuous plane thin layer coated at the sepiolite surface can block the micropores much more easily compared to nanosphere or micelle texture attached along the sepiolite surface.^17,32^ Moreover, the mesoporosity, which is related to the aggregation state of the laths, also decreased significantly after modification. This indicates that the modified sepiolites may exhibit different arrangements of the crystals compared to the unmodified sepiolite.^9,13^ However, no obvious variations of the type of aggregations among all of the clay samples can be observed by SEM, shown in Figure S4. This might be because the length and the crystal growth mechanism are the decisive factors for the arrangement of laths,^13^ and the sepiolites were fully formed before their surface modification was performed. The incorporation of the surfactant does not change any of these properties, which leads all sepiolites to tend to form similar dense meshes.

**Table 2:** Morphological Parameters Calculated from N_2_ Adsorption^a^

sample	SSA_BET_ (m^2^ g^–1^)	SSA_Ext_ (m^2^ g^–1^)	SSA_μp_ (m^2^ g^–1^)	*V*_μp,t_ (cm^3^ g^–1^)	*V*_mp,BJH_ (cm^3^ g^–1^)
pristine clay	289 (±1.4)	235	54	0.04	0.83
PANGEL B20	86 (±0.5)	86	0	0	0.12
SMC	128 (±0.2)	128	0	0	0.14
BMC	62 (±0.7)	62	0	0	0.08

a SSA_BET_ = specific surface area calculated by the BET method; SSA_EXT_ = external surface area calculated by t-method; SSA_μp_ = micropore surface area calculated by t-method; *V*_μp,t_ = micropore volume calculated by t-method; and *V*_mp,BJH_ = mesopore volume calculated by BJH method. Values in brackets represent the error estimated from the fitting procedure.

**Figure 9:**
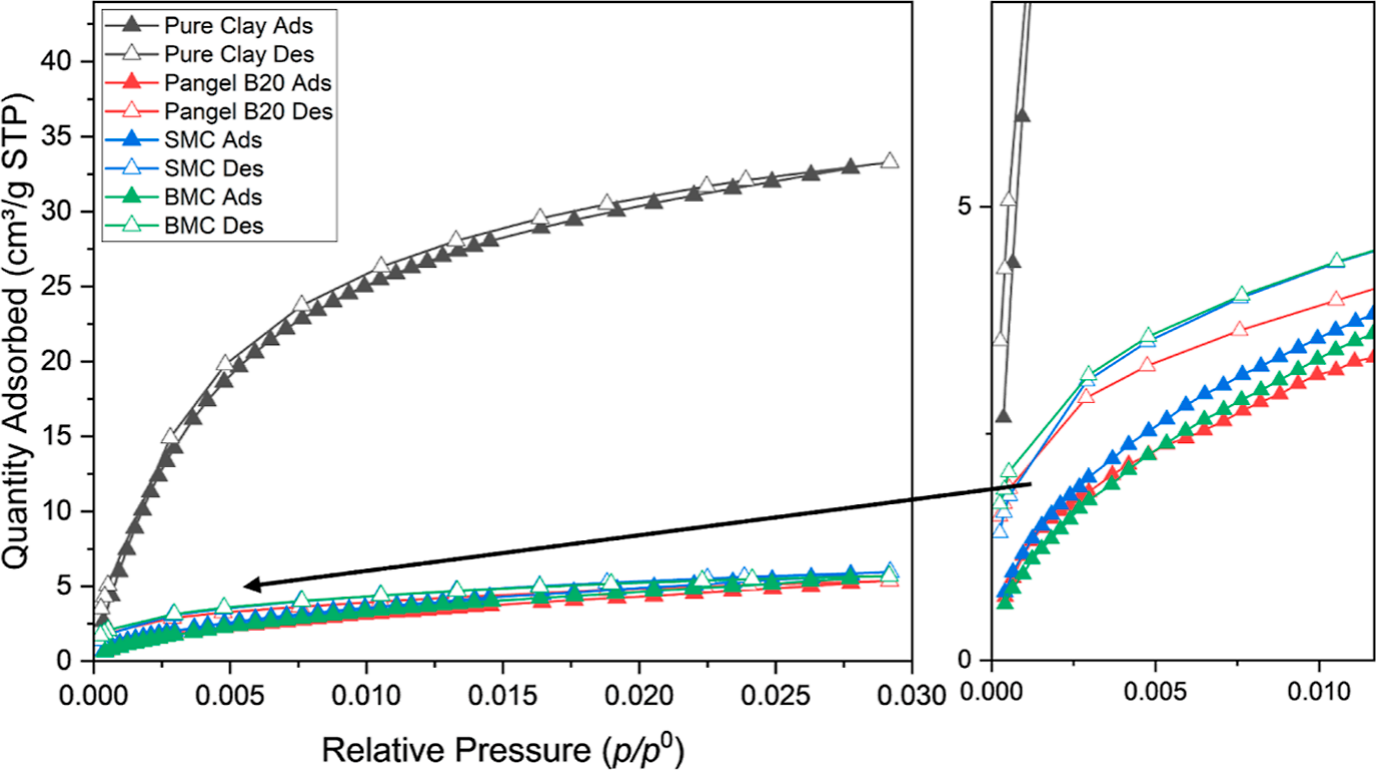
CO_2_ gas adsorption at 273 K for the four sepiolites (left) with an enlarged low-pressure region (right). Filled and empty symbols represent adsorption and desorption branches, respectively.

To further study the micropores and inner channel properties of different sepiolites, their CO_2_ adsorption/desorption isotherms were measured at 273 K, which are represented in Figure 9. The saturation pressure *p*^0^ for such temperatures is approximately 26,000 mmHg; therefore, the measurements are limited to a relative pressure up to 0.03 (corresponding to ∼1 bar).^46^ The characterizations carried out over a low relative pressure range (0–0.03) are very useful for exploring small micropores (<1 nm) such as the tunnel-shaped cavities inside sepiolites.

It is obvious that the pure sepiolite shows a much higher CO_2_ adsorption capacity compared to the other three modified sepiolites, which is consistent with *V*_μp,t_ derived from the N_2_ adsorption isotherm. This phenomenon is attributed to the fact that the surfactants hinder access to the inner microporous channels. Moreover, for all the samples, the isotherms do not appear to be totally reversible, which might be because the sepiolite can not only trap the CO_2_ physically, but chemisorption may occur, as has been reported in the literature.^52^

**Figure 10:**
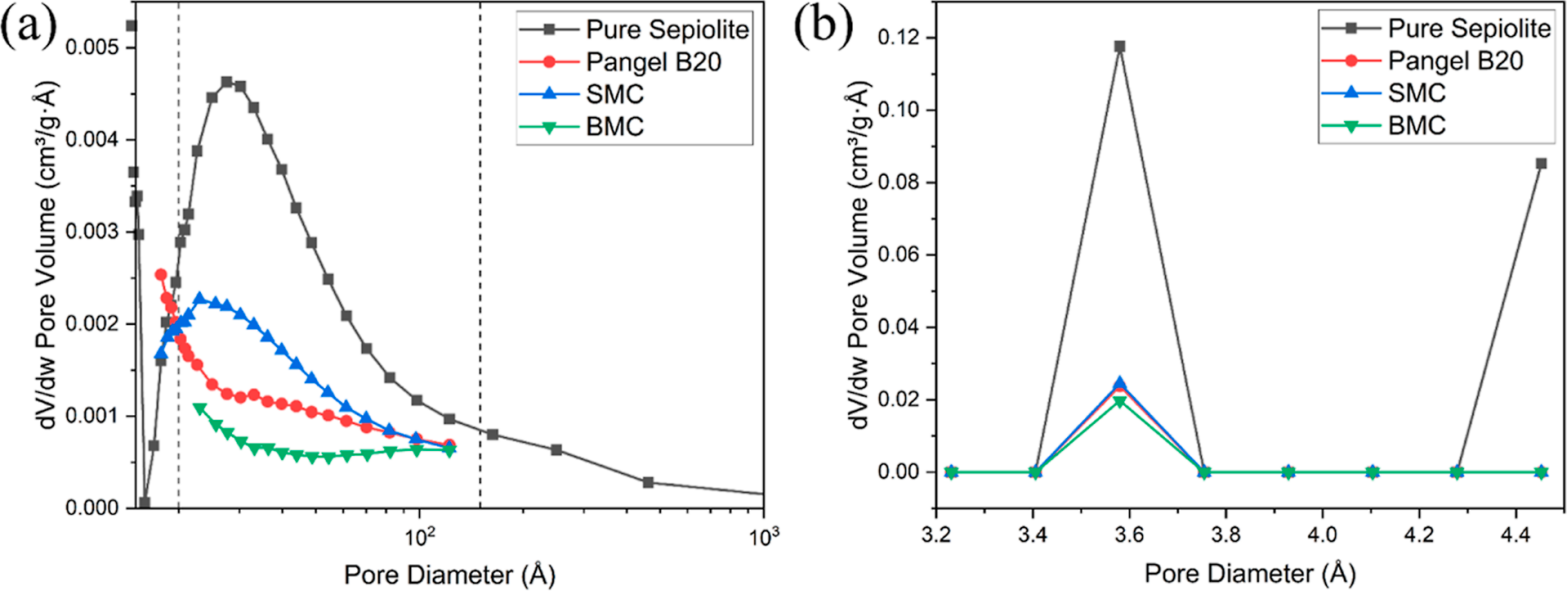
Pore size distribution of different clays extracted from (a) N_2_ adsorption at 77 K, using the BJH method; dashed line represents 20 Å (left) and 150 Å (right) and (b) CO_2_ adsorption at 273 K, using NLDFT.

Moreover, the BJH and nonlocal density functional theory (NLDFT) methods^53,54^ were used to identify the pore size distribution from N_2_ adsorption and CO_2_ adsorption isotherms, respectively. While the BJH method is only suitable for the mesopore (20–500 Å diameter) and small macropore size range (500–1500 Å diameter), NLDFT is more suitable for micropores (<20 Å diameter). In the mesoporosity and macroporosity range (Figure 10a), all clays show a characteristic peak centered around 25 Å, which can be attributed to the presence of pores due to crystal defects along the *c*-axis.^10^ Such crystal defects include stacking imperfections, variation in the width of polysomes, and omission of polysomes. Especially, the omission of polysomes can lead to commonly observed open channel defects in sepiolite particles, with the cross-sectional area from about 390 to 7500 Å^2^ for single and multiple omission, respectively.^55^ Similar pore size distributions have also been reported before.^9,56,57^ SMC, whose adsorbed surfactant has the smallest molar mass among all modifiers in this study, shows the most similar trend of distribution compared to that of pure sepiolite. PANGEL B20 and BMC display more significant differences in their pore size distribution. In the microporosity range (Figure 10b), the peaks centered at 3.58 Å are most significant, which are related to the inner channels with a theoretical width of 3.7 Å.^58^ Although all of the sepiolites show similar pore size distributions, the pure sepiolite shows a much higher pore volume compared to the other samples.

### Dispersion of Clays into a Benzoxazine Matrix

**Figure 11:**
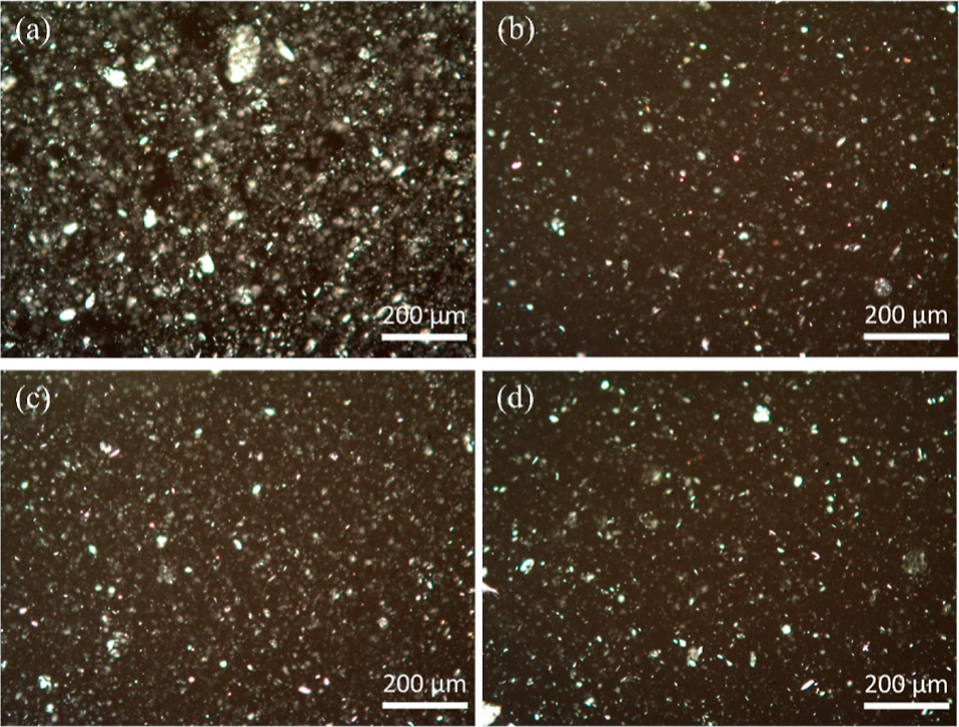
Optical microscopy images in the polarizing mode of THF/Clay/CA-a sample containing (a) pristine clay, (b) PANGEL B20, (c) SMC, and (d) BMC.

It was clearly observed (Figure 11) that all the modified clays show a much better dispersion than the native clay in the CA-a/THF mixture, which indicates a higher compatibility between the benzoxazine matrix and organophilic sepiolites. Meanwhile, our in-house prepared SMC and BMC show a similar dispersion level compared to the commercial sepiolite PANGEL B20. The prevalence of large particles (*d* > 10 μm), given in Table S4, confirms the superior dispersions achieved with modified clays and offers a quantitative comparison of the dispersion. It should be noted that the count here may only reflect the distribution of large clusters but does not consider the invisible well-dispersed nanoparticles. Generally, the large clusters of fillers inside the matrix are undesirable as they can act as stress concentrators, leading to massive microcracking and decreased mechanical properties.^59^ In conclusion, uniform dispersion of the functionalized sepiolites will undoubtedly make them more competitive enhancers for the benzoxazine matrix compared to the natural sepiolite. Moreover, the outer surface of BMC, modified using the CA-a benzoxazine, is expected to participate in the polymerization reaction during the preparation of clay/benzoxazine nanocomposites, forming a stronger linkage between the matrix and clay, as well as a more efficient force transfer bridges. Using a similar approach, Bauer et al.^60^ prepared a series of reactive nano-SiO_2_ using organosilane, and found some exhibited better surface mechanical property enhancement when incorporated in a polyacrylate matrix, compared to unmodified SiO_2_ and organophilic SiO_2_ without polymeric functionality. Furthermore, BMC may also play an important role in other resin systems such as epoxy,^61,62^ urethane,^63^ or phenolic resins,^62,64^ with which benzoxazine can copolymerize. For the potential of forming PBz/sepiolite structures based on the bespoke organophilic clays, readers can refer to Figure S5.

## Conclusions

This study demonstrates well-dispersed Bz/sepiolite blends with the ultimate aim of being used as a formulation for engineering advanced composites. Two modified sepiolites were prepared using facile and efficient methods, while a comprehensive study has been carried out on the in-house-prepared clays as well as on the pristine sepiolite and on a commercial organophilic sepiolite. The undistorted crystalline structure of the functionalized clays is evidenced by the PXRD data, while the successful addition of the surfactants is reflected by the elemental analysis and FTIR. The morphology of the sepiolites was further studied by gas adsorption and TEM–EDX, while the thermal stability was evaluated by TGA. The simple modification procedures used for SMC allow it to be used for large-batch manufacture, while the monobenzoxazine molecules used in BMC surface treatment mean it can potentially be covalently bonded to copolymeric matrices, offering optimized interfacial clay-resin matrix properties. This study represents an important step toward incorporating sepiolites as efficient mechanical enhancers in benzoxazines and other thermoset matrices.

## Supporting Information Available

Basic information on four different sepiolites, photos of dropping CA-a/THF solution into clay/water dispersion without mixing, water/sepiolite gel, XRD patterns of SMC surfactant, structure parameters of sepiolites extracted from XRD, typical dimensions of different sepiolites, atomic fraction of different elements composed of different sepiolites from EDX spectra, number of sepiolite clusters after dispersion, SEM images of sepiolite aggregation texture, and illustration of potential polymer/sepiolite nanocomposites (PDF)
