# Supplementary material for: Modified Sepiolite Nanoclays in Advanced Composites for Engineering Applications
Source: ACS Appl Nano Mater. 2024 Aug 13;7(16):19221–32. doi: 10.1021/acsanm.4c03115 (PMC11348100; doi:10.1021/acsanm.4c03115)
Supplement: Supplementary file 1 — an4c03115_si_001.pdf [file an4c03115_si_001.pdf]

Supporting Information

Modified Sepiolite Nanoclays in Advanced Composites for Engineering Applications

*Yue Tang[1], Dankun Yang[2], Valeska P. Ting[3], Ian Hamerton[4], Jeroen S. van Duijneveldt[1], Sébastien Rochat[1,4,5]**

[1]School of Chemistry, University of Bristol, Cantock's Close, Bristol, BS8 1TS, U.K.

[2]School of Electrical, Electronic and Mechanical Engineering, University of Bristol, Queen's Building, University Walk, Bristol, BS8 1TR, U.K.

[3]Research School of Chemistry, Australian National University, Canberra, ACT 2601, Australia.

[4]Bristol Composites Institute, School of Civil, Aerospace, and Design Engineering, University of Bristol, Queen's Building, University Walk, Bristol, BS8 1TR, U.K.

[5]School of Engineering Mathematics and Technology, University of Bristol, Ada Lovelace Building, Tankard's Close, Bristol, BS8 1TW, U.K.

* Email: s.rochat@bristol.ac.uk

Table S1. Basic information of four different sepiolites [a].

	Organic Modifier	Total Organic Treatment Applied	Functionalisation Mechanism	Sample Source
Pristine Clay	-	-	-	Merck
Pangel B20	Unknown	-	-	Tolsa
SMC	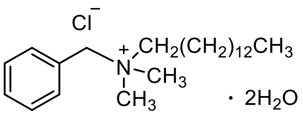	10	Cation Exchange<br>Ion-dipole Interactions	In house-Modified
BMC	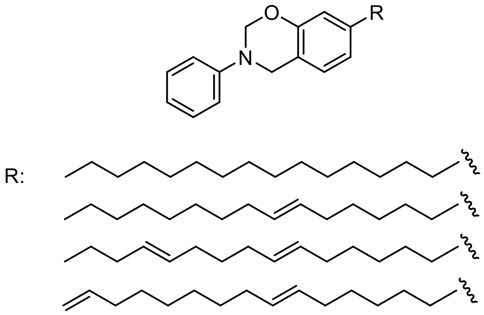	10	Physical Adsorption	In house-Modified

[a] the total organic treatment applied is the weight of surfactants added during the preparation.

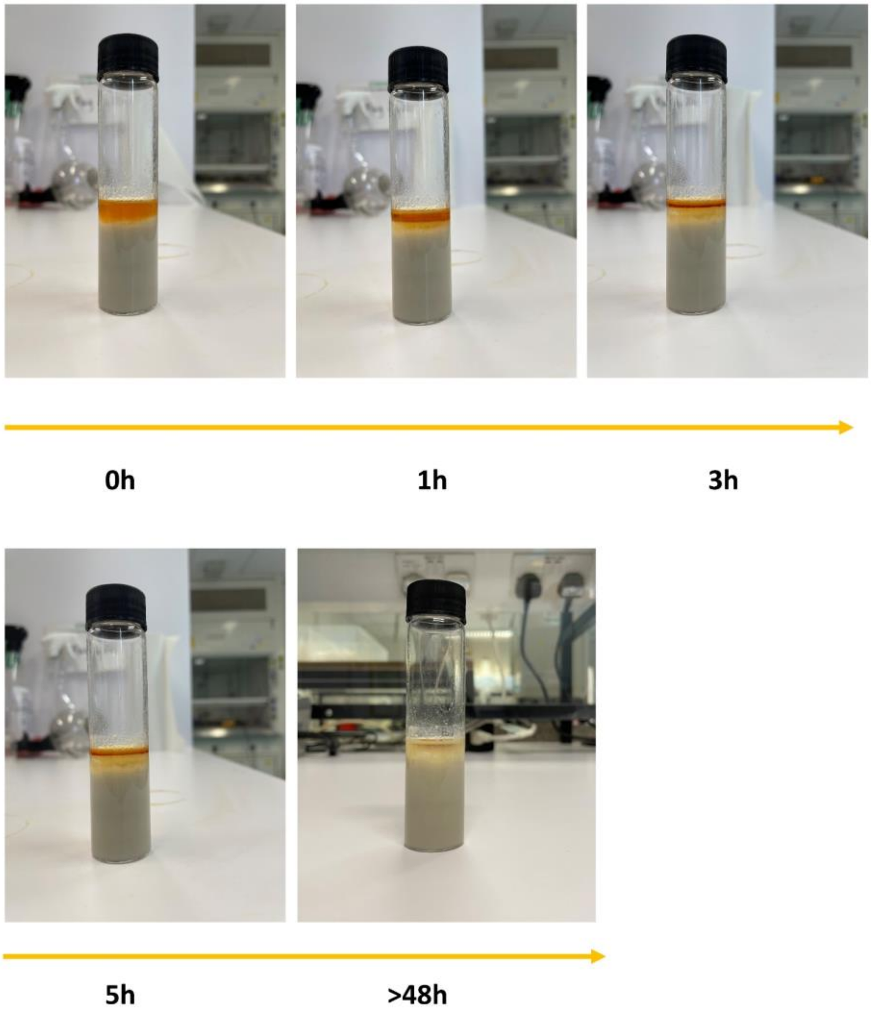

Figure S1. Drop CA-a/THF solution into clay/water dispersion without mixing.

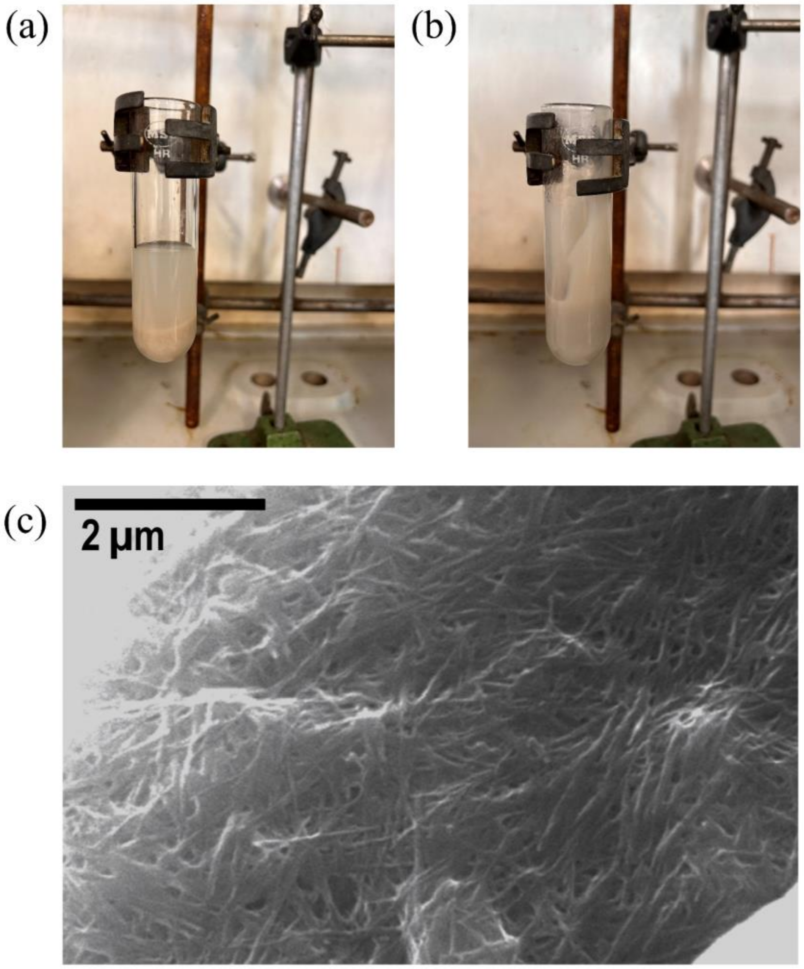

Figure S2. (a) 5 wt% sepiolite in water before high shear mixing; (b) 5 wt% sepiolite in water forming gel after high shear mixing; (c) reference microstructure of the water/sepiolite gel, reproduced with permission from García *et al.*[1]. Copyright 2011 American Chemical Society.

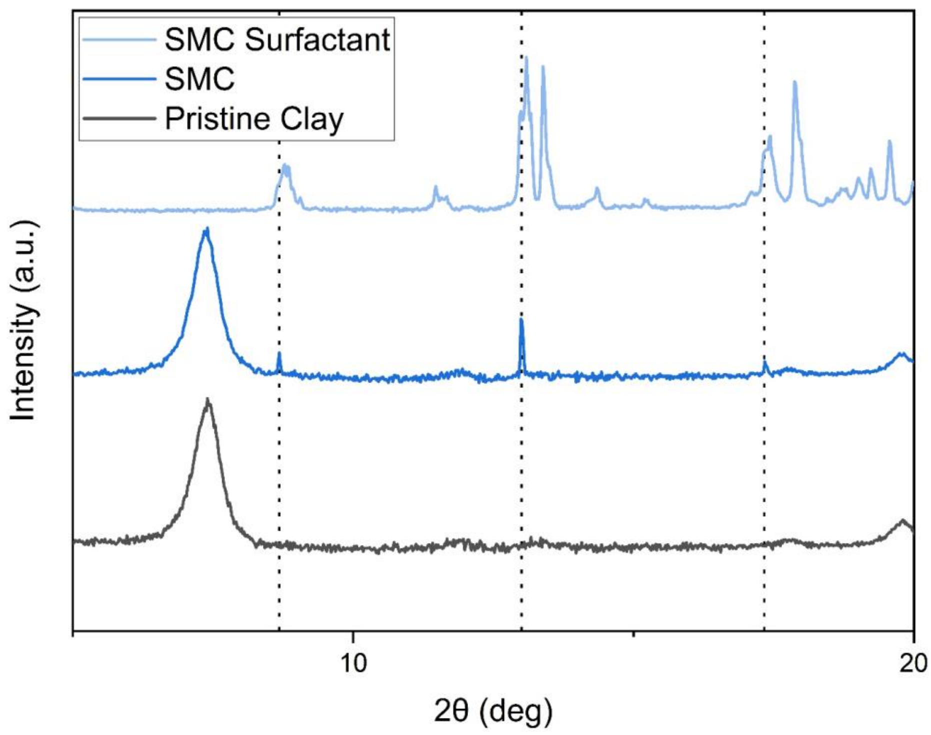

Figure S3. XRD patterns of pristine sepiolite, SMC surfactant (formulation listed in Table S1), and SMC sample in the range of 2θ (0 – 20 deg).

Table S2. Structure parameters extracted from XRD.

Sample	110 Peak Centre (deg)	130 Peak Centre (deg)	110 d (Å)	130 d (Å)
Pristine Clay	7.37	11.90	11.985	7.431
Pangel B20	7.33	11.83	12.050	7.475
SMC	7.35	11.86	12.018	7.456
BMC	7.37	11.95	11.985	7.400

Table S3. Typical dimensions of different sepiolites (10 particles account for each sample).

Sample	Dimension	
	Diameter (nm)	Length (nm)
Pristine Clay	16	478
Pangel B20	16	528
SMC	18	442
BMC	19	535

Table S4. Atomic fraction of different elements composed of different sepiolites from EDX spectra, in which dash represents undetectable.

Sample	Atomic Fraction (At%)								
	C	N	O	F	Fe	Cu	Mg	Al	Si
Pristine Clay	2.8	-	60.9	1.0	< 1.0	1.1	12.7	< 1.0	20.9
Pangel B20	7.5	-	58.6	-	< 1.0	1.0	12.4	< 1.0	19.6
SMC	12.0	< 1.0	56.1	1.2	< 1.0	1.5	10.6	< 1.0	17.7
BMC	12.3	-	55.1	-	< 1.0	1.0	10.4	< 1.0	20.5

Table S5. Number of clusters in each sample, counting based on Figure 11 (1115 μm × 836 μm).

Sample	Counts			
	d > 10 μm	d > 20 μm	d > 40 μm	d > 80 μm
Pristine Clay	354	80	11	1
Pangel B20	123	13	0	0
SMC	211	19	1	0
BMC	141	16	2	0

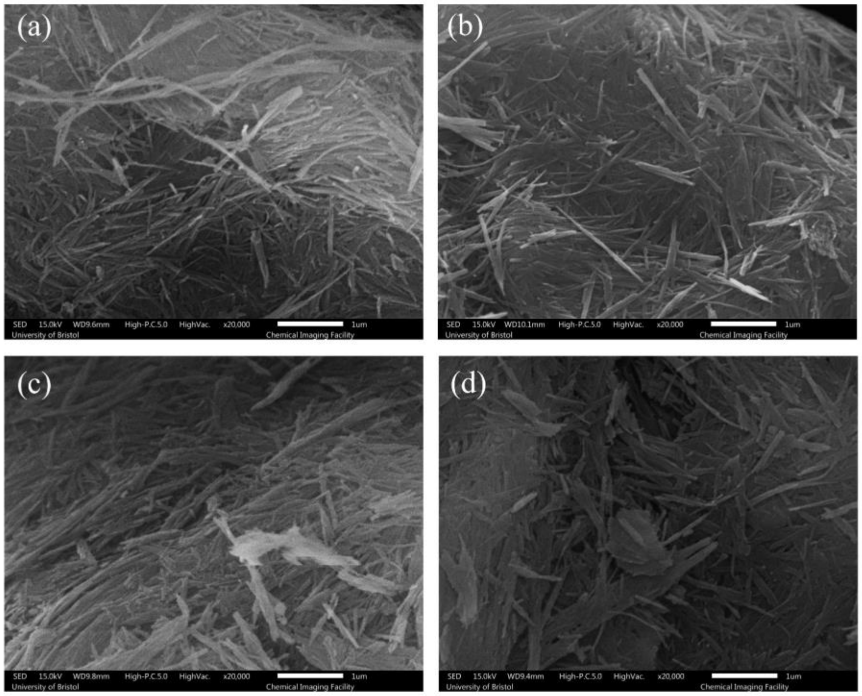

Figure S4. SEM images of sepiolites showing the typical form of aggregation texture of (a) pristine sepiolite; (b) Pangel B20; (c) SMC; (d) BMC.

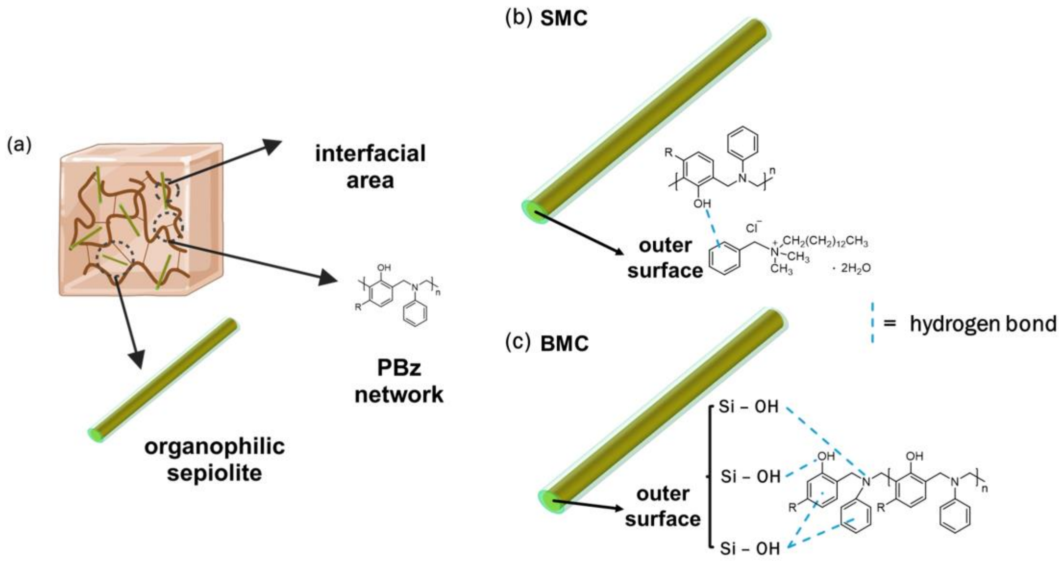

Figure S5. Illustration of (a) potentially formed polymer/sepiolite nanocomposites; (b) interfacial linkage between SMC and bulk polybenzoxazine matrix; (c) interfacial linkage between BMC and bulk polybenzoxazine matrix.

Reference

(1) García, N.; Guzman, J.; Benito, E.; Esteban-Cubillo, A.; Aguilar, E.; Santaren, J.; Tiemblo, P. Surface modification of sepiolite in aqueous gels by using methoxysilanes and its impact on the nanofiber dispersion ability. *Langmuir* **2011**, *27* (7), 3952-3959. DOI: 10.1021/la104410r.
